# Initial TK-deficient HSV-1 infection in the lip alters contralateral lip challenge immune dynamics

**DOI:** 10.1038/s41598-022-12597-4

**Published:** 2022-05-19

**Authors:** Antoine Rousseau, Oscar Haigh, Roger Legrand, Jean-Louis Palgen, Julien Lemaitre, Claire Deback, Noémie Oziol, Patrick Lomonte, Marc Labetoulle

**Affiliations:** 1 Center for Immunology of Viral, Auto-immune, Hematological and Bacterial Diseases (IMVA-HB), Commissariat à l’énergie Atomique et Aux Énergies Renouvelables, Université Paris-Saclay, INSERM U1184, Fontenay-aux-Roses, France; 2 Service d’Ophtalmologie, Hôpital Bicêtre, APHP, Université Paris-Saclay, Le Kremlin-Bicêtre, France; 3 Service de Virologie, Hôpital Paul Brousse, Université Paris-Saclay, APHP, Villejuif, France; 4 Institut NeuroMyoGène-Pathophysiology and Genetics of Neuron and Muscle (INMG-PGNM), CNRS UMR 5261, INSERM U 1513, Université Claude Bernard Lyon 1, Team Chromatin Dynamics, Nuclear Domains, Virus, Lyon, France

**Keywords:** Adaptive immunity, Infection, Inflammation, Neuroimmunology, Vaccines

## Abstract

Primary infection with herpes simplex type 1 (HSV-1) occurring around the mouth and nose switches rapidly to lifelong latent infection in sensitive trigeminal ganglia (TG) neurons. Sporadic reactivation of these latent reservoirs later in life is the cause of acute infections of the corneal epithelium, which can cause potentially blinding herpes simplex keratitis (HSK). There is no effective vaccine to protect against HSK, and antiviral drugs provide only partial protection against recurrences. We previously engendered an acute disease-free, non-reactivating latent state in mice when challenged with virulent HSV-1 in orofacial mucosa, by priming with non-neurovirulent HSV-1 (TK_del_) before the challenge. Herein, we define the local immune infiltration and inflammatory chemokine production changes after virulent HSV-1 challenge, which were elicited by TK_del_ prime. Heightened immunosurveillance before virulent challenge, and early enhanced lymphocyte-enriched infiltration of the challenged lip were induced, which corresponded to attenuation of inflammation in the TG and enhanced viral control. Furthermore, classical latent-phase T cell persistence around latent HSV-1 reservoirs were severely reduced. These findings identify the immune processes that are likely to be responsible for establishing non-reactivating latent HSV-1 reservoirs. Stopping reactivation is essential for development of efficient vaccine strategies against HSV-1.

## Introduction

Herpes simplex keratitis (HSK) is a consequence of herpes simplex virus type-1 (HSV-1) reactivation in TG neurons, and the transmission of virus anterograde to infect epithelial cells of the corneal surface. Vision loss is due to corneal damage that accumulates with each reactivation event, posing an increased threat later in life as the cumulative risk of recurrence increases with time^1–3^. Thus, suffering from HSK negatively impacts quality of life^4^. Currently, the sole strategy to reduce risk of reactivation is continuous antiviral drug intake, which reduces the frequency of recurrences by 50%^5^. Long-course treatment poses a significant increased risk of resistant strain selection^6,7^. Immunomodulatory methods for preventing HSV-1, such as by immunization, have a theoretical potential to offer protection against HSV-1 reactivation and resistance. However, a long history of attempts is yet to deliver an effective vaccine^8,9^. This may be due to the incomplete understanding of the immune mechanisms that are involved in controlling latency establishment and reactivation. Thus, there is an unmet need to explore immunomodulatory strategies that eliminate the ability of HSV-1 latent reservoirs to reactivate.

Primary human HSV-1 infection occurs in oro-facial skin for the vast majority of cases, in and around the mouth and nose^1,10^, in childhood and early during adult life^11^. In very rare cases where primary corneal infection occurs, it appears as diffuse micro-dendritic ulcerations^10^. Whereas, clinical HSK is caused by reactivation of already established latent reservoirs in the TG.

Skin is the natural site of primary infection, and this tissue bears unique properties to trigger immune responses to HSV-1^12,13^. Innate immune cells and inflammatory responses provide a resistance to HSV-1 replication early after infection^14–20^. Adaptive responses are facilitated by dendritic cells (DC)^13,21^ to provide long-lasting memory that can eliminate active HSV-1 replication, but take time to develop^22^. The delay, and virulence factors employed by HSV-1 prevent prophylactic immunity^23,24^, so HSV-1 progeny rapidly infect neurons of the trigeminal ganglia (TG)^25,26^. Replication here enables the spread of virus to additional neurons within the TG. However, the viral life cycle is self-limiting, rapidly switching to the latent state (infectious particles are no longer produced^27^). Latent viral genomes persist as a reservoir for potential reactivation in TG neuron nuclei, with the ability to reactivate for the life of the host.

Our model of HSV-1 infection in mice is appealing as it reproduces most aspects of natural HSV-1 infection in humans, by preserving the natural site of primary infection (inoculation occurs in the lip, to produce a localised lesion on one side of the mouth). Acute phase unilateral HSK develops, while latent HSV-1 reservoirs establish bilaterally^28–30^. Priming the orofacial mucosae with a non-neurovirulent HSV-1 mutant (TK_del_) in the same fashion, before virulent contralateral lip challenge, elicits complete protection against HSK (and other acute signs of the disease) if a delay of 4 days is respected between the prime and the challenge^30^. Furthermore, reactivation of latent TG HSV-1 reservoirs in protected mice are not triggered by explant culture (a standard technique that provokes reactivation^31,32^).

To explore the underlying immune effectors, we characterized infiltrating immune cells, the local presence of inflammatory chemokines, and viral gene expression at important infection sites after virulent challenge of naïve vs TK_del_-primed mice. Significant shortcomings in the immune responsiveness of naïve mice were revealed upon challenge, whereas TK_del_-primed animals were endowed with heightened immunosurveillance and rapid lymphocyte-rich immune responses that associated with tempered local inflammation and attenuated gene expression in the TG. These findings describe specific immune changes that are linked to the establishment and maintenance of a ‘dead end’ latent state, which is unable to reactivate when triggered by explant culture, elicited by non-neurovirulent HSV-1 priming. These results provide a basis for defining immune mechanisms that might be exploitable for invoking immunity to HSV-1-mediated disease, by inhibiting reactivation of latent reservoirs.

## Methods

### Virus strains and cells

Virulent patient-derived WT strain SC16^33^ has been maintained at low passage number and plaque purified, and mutant TKDM21 (termed TK_del_, a kind donation from Stacey Efstathiou) were propagated and titrated as previously described^28^. TK_del_ contains an 816-bp deletion of the thymidine kinase coding region^34^, which makes it non-neurovirulent. We confirmed viral replication in the lip and iTG by SC16, but only in the lip with TK_del_ by plaque assay of inoculated lip tissue or iTG non-denatured lysates, on indicated days during acute infection (Supplementary Fig. S1).

### Inoculation procedures

**Figure 1:**
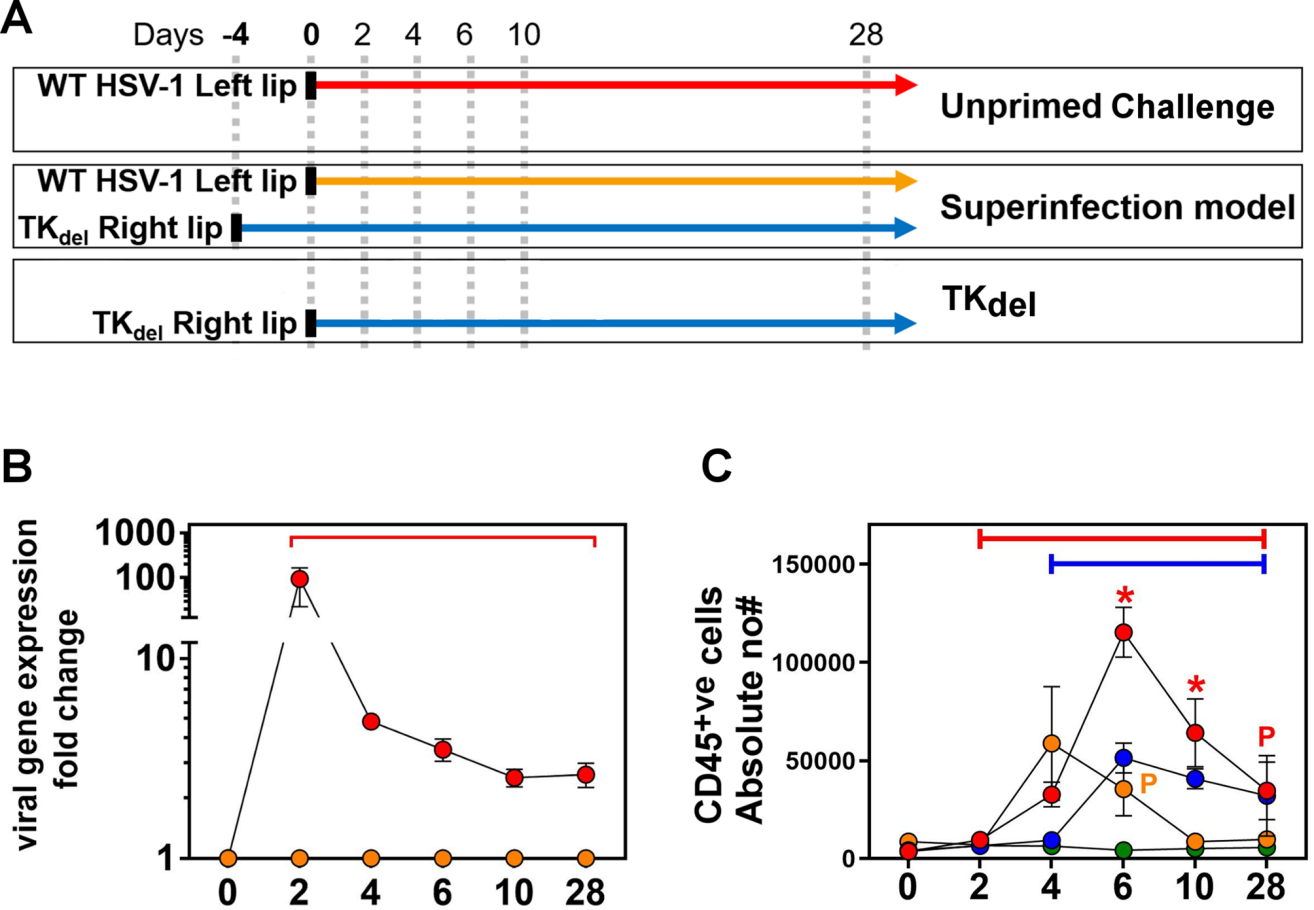
Infection regime, WT HSV-1 gene expression, and immune cell infiltration in the iTG. Schematic of the superinfection model, versus the challenge of unprimed (naïve) mice (**A**). Naïve mice were infected in the lip with either PBS (Mock), WT HSV-1, or with TK_del_ on day 0. Superinfection model mice were initially infected with TK_del_ in the right lip on day -4 and then challenged with WT HSV-1 on day 0 in the left lip. On different days for each group, lip and TG were harvested from individual mice, and WT HSV-1 thymidine kinase gene expression was measured from TG by RT-qPCR (**B**), while CD45^+ve^ cells were defined and absolute numbers enumerated by flow cytometry (**C**). Each point represents *n* = *4* individual biological replicates. *Colored bars* indicate when the color-matched group was significantly different to uninfected day 0 (*p* < *0.03; permutation test*). *Red asterisks* identify times when WT HSV-1 was significantly different to PBS mock infected (green) and superinfection model. *Orange asterisks* indicate significant difference between superinfection model versus infection in unprimed mice (permutation test, *p* < *0.03*). A significant difference between mock and only the color-corresponding group is indicated with a *colored P*. *Figures were formulated using Flowjo, Excel and Prism*.

Female BALB/c mice at 6 weeks of age were always infected with 1 × 10^6^ PFU of virus diluted in 1 µL of sterile PBS, as defined in^28^. Mice were inoculated with TK_del_ (Superinfection model) or PBS (unprimed) in the right lip, and 4 days later, all mice were inoculated with WT HSV-1 in the left lip for virulent challenge (Fig. 1A). Mice were sacrificed randomly on days 2, 4, 6, 10 (acute phase), and day 28 (corresponding to latent infection in our model^35^). TK_del_ infection alone was performed in the right lip of additional mice, which were sacrificed at the same time points (TK_del_). Day 4 in the TK_del_ group corresponds to day 0 of the superinfection model. Untreated mice were sacrificed at day 0 for negative controls.

All procedures conformed to the Association for Research in Vision and Ophthalmology (ARVO) ethical standards statement for the use of animals in research, were in performed compliance with ARRIVE guidelines and were also approved by the institutional ethics committee “*Comité d’éthique en matière d’expérimentation animale Paris Centre et Sud*” (CEEA 59).

### Sample collection

Specimens of the upper left and right lip, approximately 2 × 2 × 2 mm and encompassing the site of inoculation were harvested just internal to the labial commissures, after which left and right TG were harvested from the same animals by the same surgeon.

### Detection of immune infiltrate by multiparameter flow cytometry

Single cell suspensions were prepared by mincing with fine scissors, and digestion with collagenase IV (Scima/Worthington Chemicals) and DNase I from bovine pancreas (Sigma-Aldrich)^36,37^. Myelin was removed using 37% Percoll Plus (GE Healthcare) gradient^37^. Cells were labelled in PBS using the same fluorescently conjugated antibodies and concentrations (against CD45-BV605, Ly6G-AF700, CD11c-BV785, CD11b-APC-Cy7, IA/IE-BV650, CD24-BV711, CD64-BV421, and Ly6C-PercpCy5.5, and Live/Dead fixable Aqua), sourced from the same providers previously described by Yu et al*.*^38^. Also included were: anti-mouse CD3-PE-Cy7 (Fischer Scientific), anti-mouse CD4-BUV395 (BD Biosciences) and anti-mouse CD8α-PE-CF594 (BD Biosciences). True count beads were also used, according to manufacturers’ specifications to determine absolute cell numbers of cells with the following formula: [(no. of events)/(no. of bead region events] × [(total no. of absolute-count beads)/(test volume [300 μL])]. Acquisition was performed using a 5-laser 20-filter FORTESSA X20-flow cytometer (Becton Dickinson). Immune populations, including: inflammatory monocytes, neutrophils, NK cells, eosinophils, macrophages, CD11b^+ve^ DC, CD11b^−ve^ DC and B cells were defined using the strategy previously published by Yu et al*.* (Supplementary Fig. S2 and^38^). Addition of a single cell gate, bead gating, and anti-CD3 anti-CD4 and anti-CD8 antibodies were incorporated to further define CD4^+ve^ and CD8^+ve^ T cells, and double negative lymphocyte populations. Acquisition of the total of each sample was performed, which yielded on average 7 × 10^5^ ± 2 × 10^5^ cells per sample.

### Detection of WT HSV-1 viral gene expression by RT-qPCR

Tissue samples were collected in RNAlater and stored at 4 °C overnight. Excess RNAlater was removed, and tissue transferred into RLT buffer containing β-mercaptoethanol (QIAGEN). Homogenization occured in CKmix tubes (BertinPharma) at 6500 RPM for 45 secs using a Precellys24 (BertinPharma). RNA was extracted using RNAeasy mini kit (QIAGEN). DNA was digested using Machrey Nagel rDNAse and cDNA synthesized from RNA by SuperScript™ VILO™ (Thermo Fischer Scientific) reaction. Primers for WT HSV-1 thymidine kinase cDNA were designed targeting the deleted sequence of the TK_del_ mutant^34^ (F: GTGGTAATGACAAGCGCCCA, R: GGGGTCATGCTGCCCATAAG), also RPL13α housekeeping gene primers (F: GTGGTCGTACGCTGTGAAGG, R: CCTCGGGAGGGGTTGGTATT) were made (Eurofins Genomics). QuantiFast SYBR Green PCR Kit (QIAGEN) was used for quantitative PCR. After hot start 5 min at 95 °C, cycling involved: 10 s at 95 °C and 30 s at 60 °C for 40 cycles followed by melt curve. All kits and reagents were used according to manufacturers’ specifications.

### Bead-based immunoassay for quantification of inflammatory chemokines

Individual tissue samples were collected in 0.5 mL of non-denaturing × 4.7 conc lysis buffer (Cell Signaling Technologies), with 1 mM phenylmethylsulfonyl fluoride (Sigma-Aldrich) added immediately prior to harvesting. Tissues were homogenized as described for RT-qPCR. Homogenates were clarified at 10,000×*g* for 5 min at 4 °C and frozen at − 80 °C. Mouse proinflammatory chemokines LEGENDplex assay (Biolegend) was performed with twofold-diluted samples. Data acquisition was performed using a FORTESSA X20 flow cytometer (Becton Dickinson) to acquire 4 000 beads/sample.

### Data and statistical analyses

LEGENDplex v.8.0 software (Biolegend, http://www.vigenetech.com/) and Excel (Microsoft Office Professional Plus 2016, https://www.microsoft.com/) were used to calculate chemokine concentrations. Standard curves achieved r > 0.995, and detected levels were within standard curve range. Cytometric data was analyzed using Flowjo v10.0 (https://www.flowjo.com/). Raw data was analyzed using Excel. Figures were generated using Prism v7.00 (Graphpad, https://www.graphpad.com/) and GIMP v2 (https://www.gimp.org/). Statistical analysis was performed with R version 3.2.0. (The R Foundation, https://www.r-project.org/). Cell numbers or viral gene expression values were compared using the exactRankTests permutation test, and chemokine concentrations using the Student’s t-test. Statistical significance was indicated by p < 0.03 (2-tailed).

## Results

### Reduced challenging HSV-1 transcription and immune infiltration in iTG of primed mice

Unprimed mice were challenged with virulent WT HSV-1 in the left lip, and compared to mice that were primed in the right lip with TK_del_ 4 days before challenge in the left lip with WT HSV-1 (Fig. 1A). These groups were also compared to mice that were challenged with TK_del_, or with PBS. Gene transcription of the virulent WT challenge parental HSV-1 was monitored specifically by quantitative RT-PCR targeting the deleted sequence of the TK_del_ mutant (Fig. 1B). After lip challenge, WT HSV-1 gene expression was already prolific in the TG ipsilateral (iTG) of naïve mice 2 days post challenge (94-fold increase from baseline), and diminished thereafter, ultimately entering a slightly elevated plateau until day 28. In comparison, WT HSV-1 gene expression was not detected in the iTG after the challenge of TK_del_-primed mice for the entirety of the study period (until day 28). These data demonstrate the immunity elicited by priming with TK_del_, to restrict virulent challenge viral gene expression in the TG and suggests an absence of lytic infection.

Absolute immune cell (CD45^+ve^) numbers increased subtly (2.5-fold) by day 2 post infection in the TG ipsilateral (iTG) to lip infection in unprimed mice (Fig. 1C), 8.6-fold by day 4, and peaked 30-fold higher by day 6. CD45^+ve^ cell numbers started to decline by day 10, but persisted higher than baseline (ninefold) at day 28. Upon virulent challenge in the superinfection model, a similar increase in CD45^+ve^ cell numbers occurred until day 4 in the iTG, compared to unprimed mice. However in striking contrast, this was the peak of infiltrate. By day 6 CD45^+ve^ infiltrates had declined significantly (compared to the unprimed group: *p* =  < *0.03*). By day 10, CD45^+ve^ cell numbers had receded and remained at baseline to day 28.

Combined, these data demonstrated a slow-reacting iTG infiltration by immune cells in challenged unprimed mice, which started to arrive after fulminant viral gene expression, and persisting into the latent phase of infection. In contrast, priming with TK_del_ resulted in undetectable superinfecting WT HSV-1 gene expression, and a reduced magnitude of infiltrate that subsides back to baseline levels during acute phase.

### Acute inflammatory cells and latent-persisting T cells lost from iTG after prime-challenge

**Figure 2:**
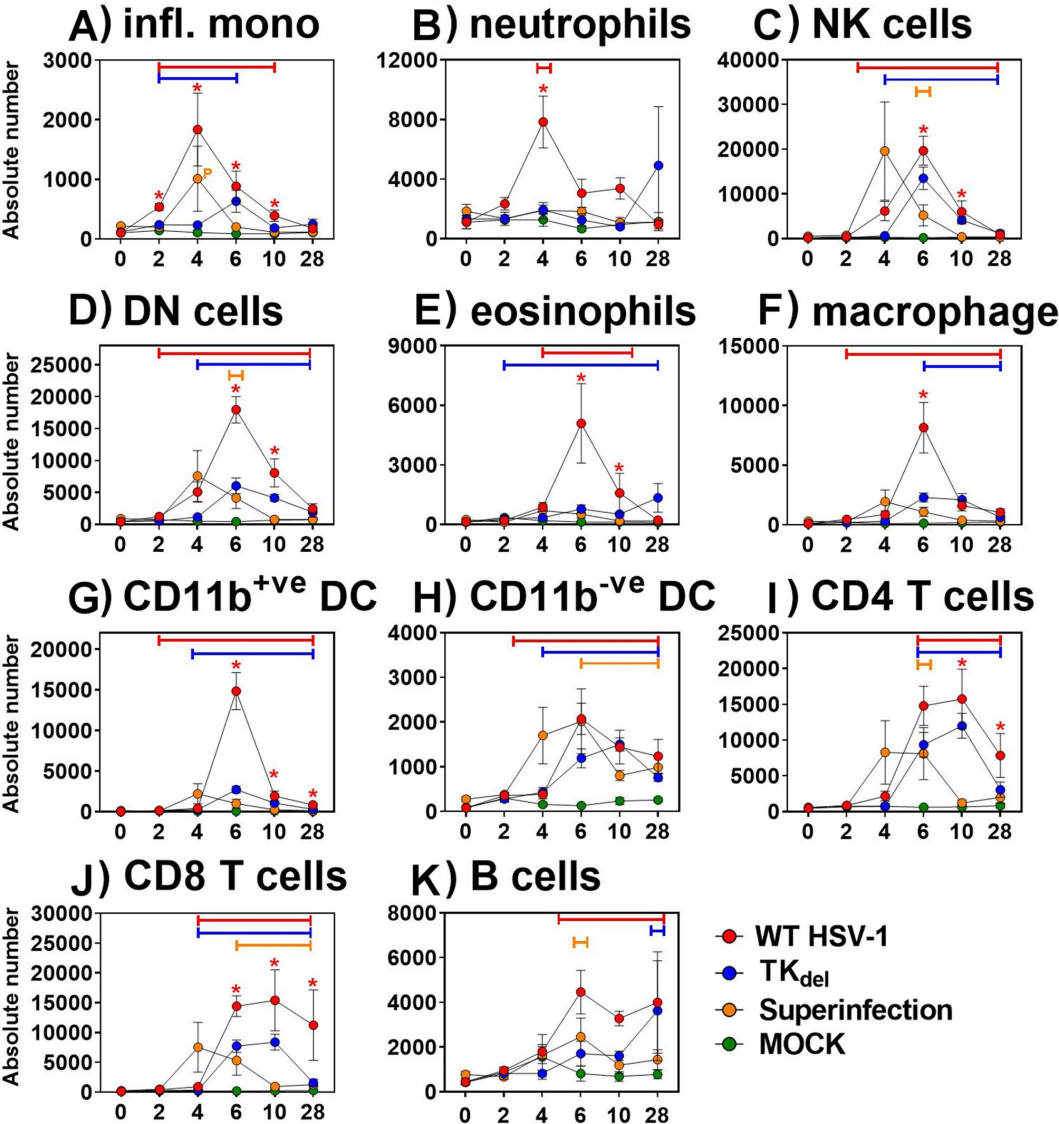
Immune subsets in the iTG. Naïve mice were infected in the lip with WT HSV-1, PBS (mock; green line), or with TK_del_. Mice of the superinfection model received an initial inoculation of TK_del_ in the right lip on day -4, and were challenged with WT HSV-1 on day 0 in the left lip. On different days for each group, immune subsets (**A**–**K**) were defined and absolute numbers quantified by flow cytometry in iTG single cell suspensions. *Colored bars* indicate significant differences from uninfected tissue at day 0. *Red asterisks* identify when WT HSV-1 compared to PBS mock infection (green) and superinfection model was significantly different (*p* < *0.03; permutation test*). *Colored P* indicates significance between Mock and the color-corresponding group. Each point represents *n* = *4* individual biological replicates. *Figures were formulated using Flowjo, Excel and Prism.*

In unprimed mice that were challenged in the lip with virulent WT HSV-1, inflammatory monocytes appeared in the iTG on day 2 post challenge, to peak on day 4 (Fig. 2A). Neutrophil infiltrate peaked on day 4 (Fig. 2B). NK and DN T cell influx began by day 4, peaking at day 6 (Fig. 2C,D), when a spike in eosinophils, macrophages, CD11b^+ve^ and ^−ve^ DC, CD4 and CD8 T cells, and B cell numbers occurred (Fig. 2E–J). Infiltration subsided by day 10, with the exception of CD11b^−ve^ DC, CD4 and CD8 T cells, and B cells (Fig. 2H–K). At day 28, significant numbers of T cells (the greatest immune component at this time (Fig. 2I,J)), macrophages (Fig. 2F) and CD11b^−ve^ DC (Fig. 2H) remained in the iTG.

In contrast, subsets were reduced or absent from infiltrates in iTG of TK_del_-primed mice after virulent WT challenge. Inflammatory monocyte infiltrate was reduced and delayed, appearing by day 4 (Fig. 2A). Furthermore, the spike in neutrophils, DN T cells, eosinophils, macrophages, and CD11b^+ve^ DC numbers were absent from challenged TK_del_-primed mice (Fig. 2B,D–G); similarly, they were minimal/absent in mice after prime with TK_del_. Most immune subsets that increased significantly after prime with TK_del_did so on day 6 (NK, DN, CD11b^−ve^ DC, CD4 and CD8 T cells, and B cells; Fig. 2C,D,H–K). Interestingly, on day 28 only modest increases in CD4 and CD8 T cells (Figs. 2I,J) were observed in the iTG of TK_del_-primed mice that were challenged.

These data demonstrated differing immune landscapes in the iTG of TK_del_-primed vs naïve mice upon infection with virulent WT HSV-1, with the reduction or absence of key inflammatory immune subsets during acute infection and absence of classical T cell persistence in latent HSV-1 TG reservoirs, by priming animals with TK_del_ before challenge.

### Absence of inflammatory chemokines in iTG of TK_del_-primed mice after challenge

**Figure 3:**
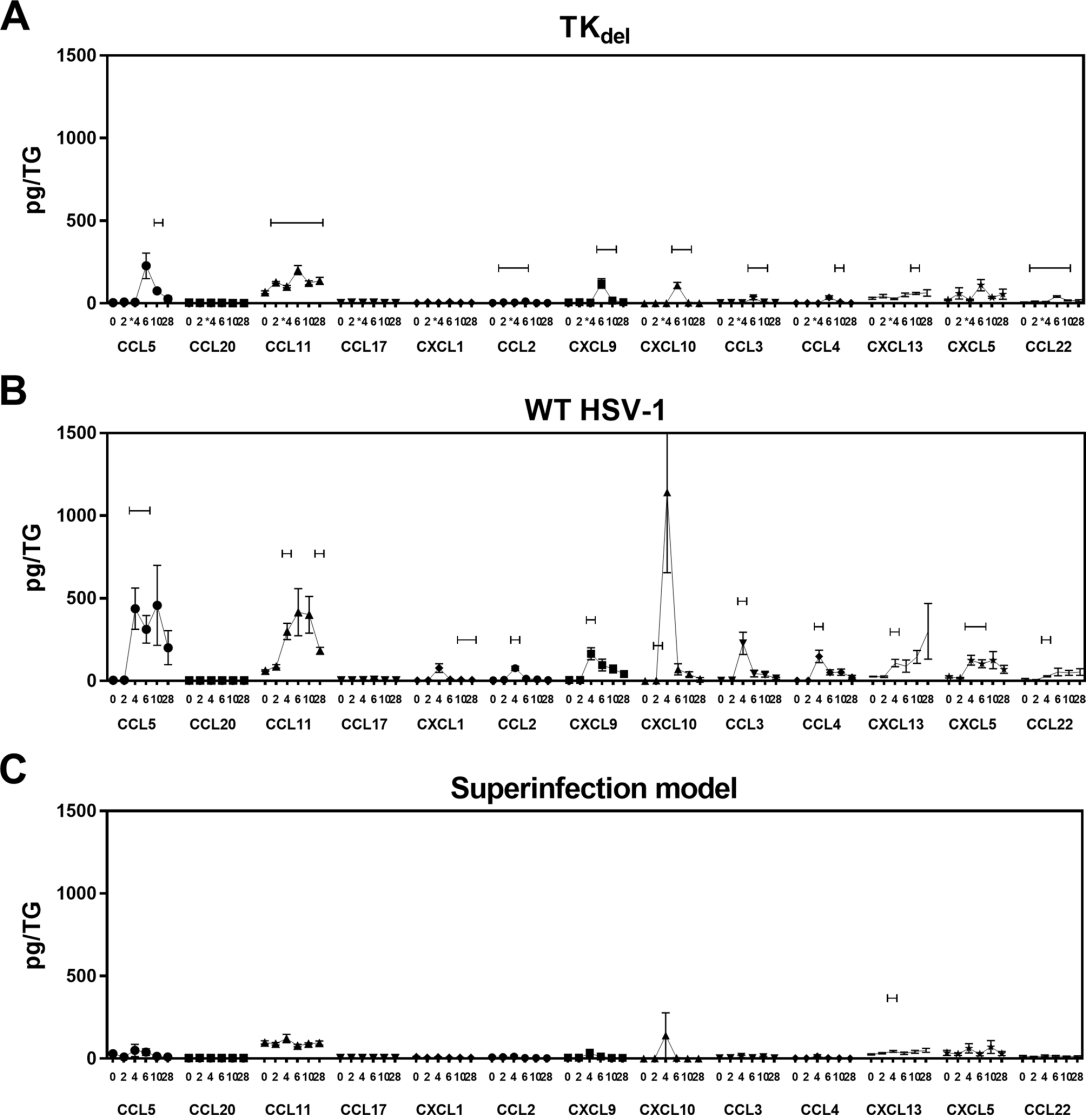
Inflammatory chemokines in the iTG. Naïve mice were infected in the lip with TK_del_ (**A**), or WT HSV-1 (**B**) on day 0. Superinfection model mice were initially infected with TK_del_ in the right lip on day -4, and then challenged with WT HSV-1 in the left lip on day 0 (**C**). Inflammatory chemokines in non-denatured iTG lysates were quantified using multi-analyte flow cytometry assay. Bars indicate significant difference from uninfected day 0 (*p* < *0.03; T-test*). Each point represents *n* = *4* independent biological replicates. *Figures were formulated using LEGENDplex software, Excel and Prism.*

Following challenge with TK_del_ or WT HSV-1, inflammatory chemokines increased in the iTG by days 4 or 6 (Fig. 3A,B, respectively). CXCL10, and to a lesser extent CCL5 and CCL11, had the greatest increases during acute infection. However, levels were lower and delayed overall in the iTG of TK_del_-challenged mice. In the iTG of mice primed with TKdel and then challenged with virulent WT, most inflammatory chemokines were not detected (Fig. 3C), except for CXCL13 on day 4. These data demonstrate attenuation of inflammatory conditions in the iTG of TK_del_-primed mice upon virulent challenge, compared to challenged naïve mice.

### Rapid infiltrate and impaired viral transcription in challenged lip elicited by TK_del_ priming

**Figure 4:**
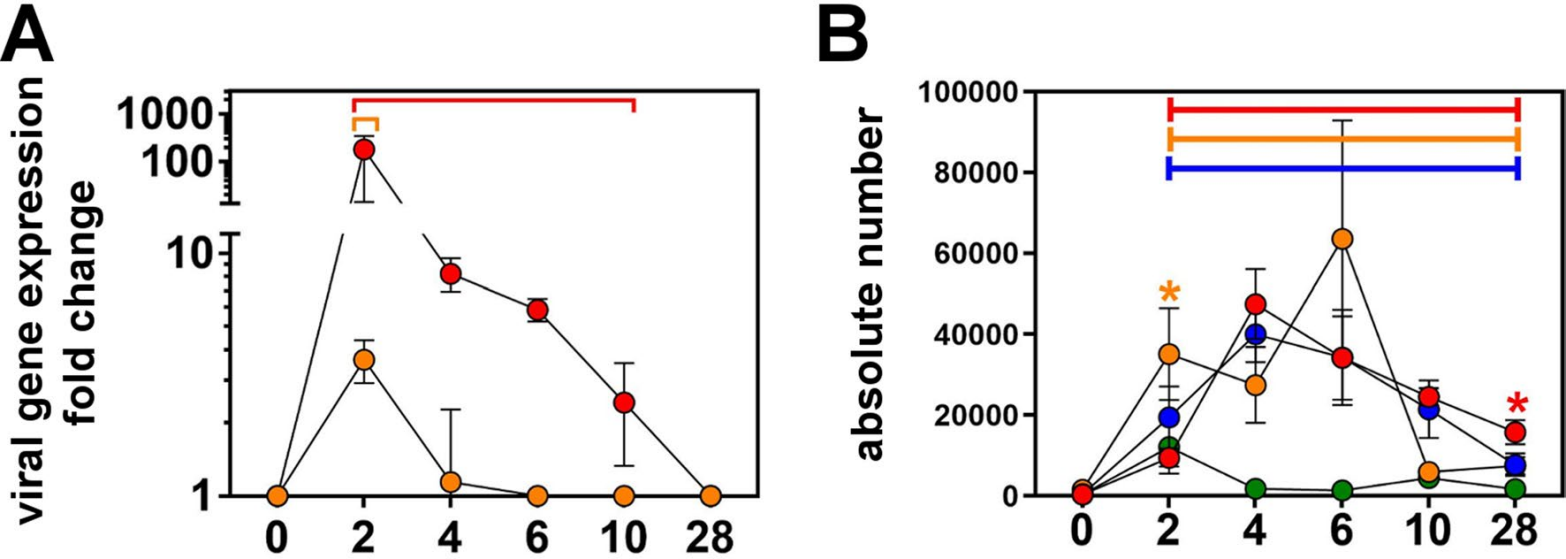
WT HSV-1 gene expression and immune infiltrate at the lip inoculation site. Naïve mice were infected in the lip with PBS (Mock; green), WT HSV-1, or TK_del_ on day 0. Superinfection model mice were initially inoculated with TK_del_ in the right lip on day -4 and then superinfected with WT HSV-1 on day 0 in the left lip. On different days for each group, thymidine kinase viral gene expression was measured by RT-qPCR in inoculation site lip tissue (**A**), while CD45^+ve^ cells were defined and absolute numbers enumerated from single cell suspensions by flow cytometry (**B**). *Colored bars* indicate significant differences from uninfected tissue at day 0 (permutation test, *p* < *0.01*), while *asterisks* indicate significance between both unprimed WT HSV-1 and Mock (green) versus superinfection model (*orange*), or both superinfection model and PBS mock infection versus unprimed WT HSV-1 (*red*) (*p* < *0.03; permutation test*). Each point represents *n* = *4* individual biological replicates. *Figures were formulated using Flowjo, Excel and Prism.*

Compared to uninfected day 0, viral gene expression at the lip inoculation site for the virulent WT challenge in naïve mice peaked at day 2 (179-fold), drastically reducing by day 4 (eightfold), and was extinguished by day 28 (Fig. 4A). In contrast, WT HSV-1 gene expression on day 2 at the site of lip challenge in TK_del_-primed mice increased only 3.7-fold. This was 48-fold lower than the challenge of naïve mice, and was almost undetectable by day 4.

In naïve mice, immune infiltrates at the site of virulent WT lip challenge increased until day 4, and had partially receded by day 28, to remain about 40 times greater than immune cell numbers in mock-infected lips (Fig. 4B). In contrast, an early surge of immune infiltrate occurred at the site of lip challenge, on day 2, in mice that were primed with TK_del_, which was threefold greater than day 2 lip infiltrate in challenged naïve mice. Thereafter, CD45^+ve^ cells in the superinfected lip fluctuated, but decreased to mock-infected levels by day 10. These data demonstrated that priming with TK_del_ elicited more rapid immune infiltration of the virulent WT challenge site (the contralateral lip), which was associated with better control of viral gene expression.

### Priming enhanced lymphocytic infiltration of the challenged lip

**Figure 5:**
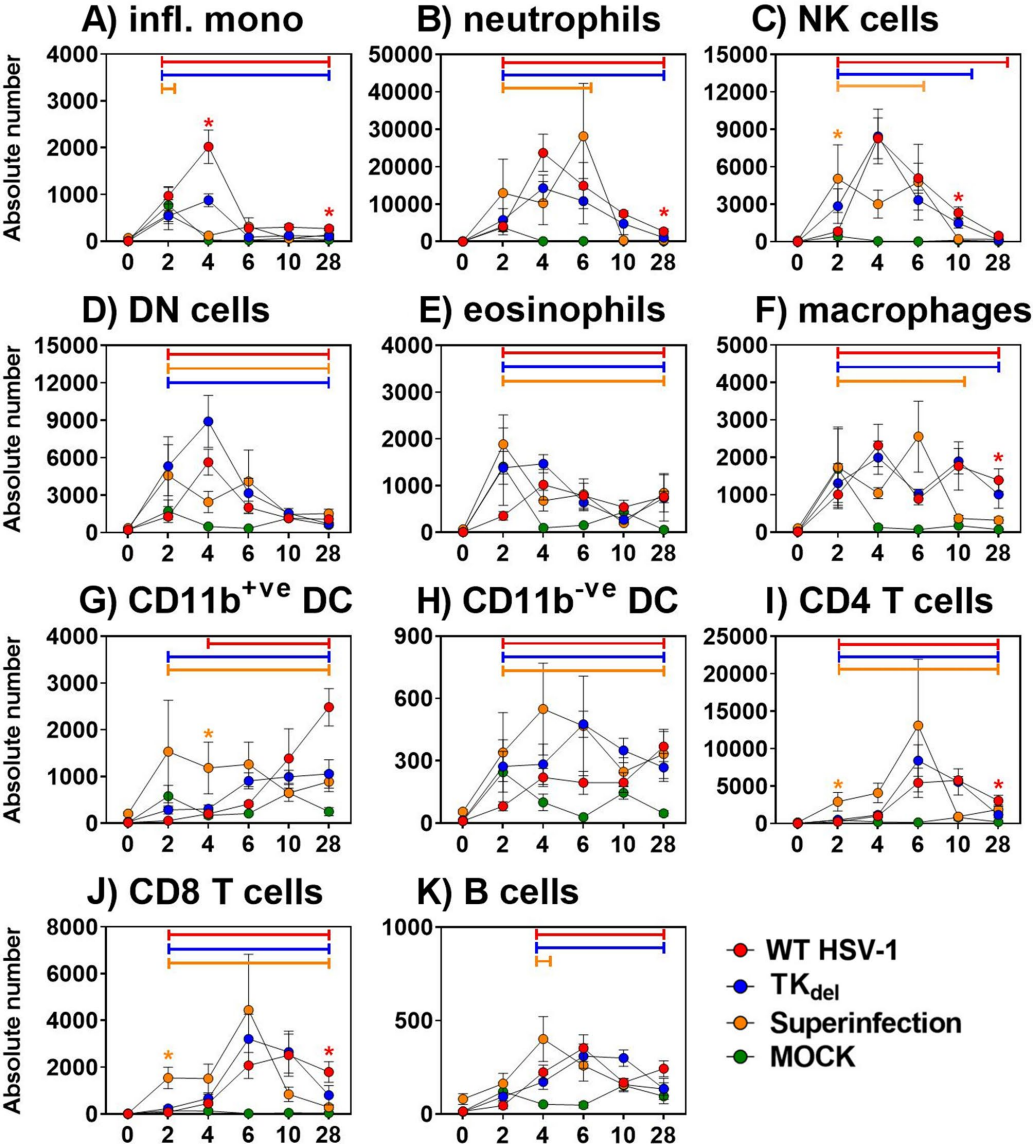
Immune subsets at the lip site of inoculation. Naïve mice were infected on day 0 with WT HSV-1, PBS (mock), or TK_del_. Mice of the superinfection model received an initial inoculation of TK_del_ in the right lip on day -4, and were superinfected on day 0 in the left lip with WT HSV-1. On different days in each group, immune subsets (**A**–**K)** were defined and absolute numbers quantified by flow cytometry from single cell suspensions of the lip site of virus inoculation. *Colored bars* indicate significance from uninfected tissue on day 0. *Red asterisks* indicate a significant difference for unprimed WT HSV-1-infected mice compared to PBS mock infection (green) and superinfection model mice, and *orange asterisks* indicate significance for superinfection model compared to Mock and WT HSV-1, and (*p* < *0.03; permutation test*). Each point represents *n* = *4* individual biological replicates. *Figures were formulated using Flowjo, Excel and Prism.*

On day 4, an inflammatory monocyte influx appeared at the virulent WT challenge lip site in naïve mice, which did not develop in the challenged lip of TK_del_-primed mice (Fig. 5A). This population persisted, at low yet significant levels, in the challenged lip site of naïve mice up to day 28. Neutrophils were the greatest infiltrating population, peaking on day 4-post challenge and remaining elevated until day 28 in the WT-challenged lips of unprimed mice. Neutrophils returned to baseline in TK_del_-primed animals by day 10 after WT challenge (Fig. 5B). While macrophage infiltrates at the site of all HSV-1 challenges reached similar magnitudes during early acute time points (days 2–6), they declined to baseline levels on day 10 (and remained at this level until day 28) only in the WT-challenged lip of TK_del_-primed mice, remaining elevated in unprimed mice until day 28 (Fig. 5F). There was a slightly elevated presence of CD11b^+ve^ DC in the challenged lip of the superinfection model (Fig. 5G). DN T cells, eosinophils, CD11b^−ve^ DC and B cells also infiltrated the WT-challenged lip in all groups, but without a discernible difference between infection regimes (Fig. 5D,E,H,K). NK cells, CD4 and CD8 T cells, infiltrated the WT-challenged lip of TK_del_-primed mice more rapidly, being much higher on day 2 (respectively 3-, 13- and 19-fold) than near-baseline levels in the WT-challenged lip of naïve or mock-challenged mice (Fig. 5C,I,J; *p* < *0.03 for all conditions*). These earlier infiltrates had also decreased more rapidly, by day 10, with very few NK or T cells persisting at day 10 or day 28. Thus, from the earliest point tested there was an enrichment of NK cells, CD4 and CD8 T cells, a greater abundance of CD11b^+ve^ DC, and reduced innate inflammatory subsets at the site of virulent WT HSV-1 challenge in the lip, when mice were primed with TK_del_. In contrast, infiltration of the WT-challenged lip in naïve mice was slower, and with greater subset persistence up to day 28.

### Inflammatory chemokines reduced in the superinfection model lip

**Figure 6:**
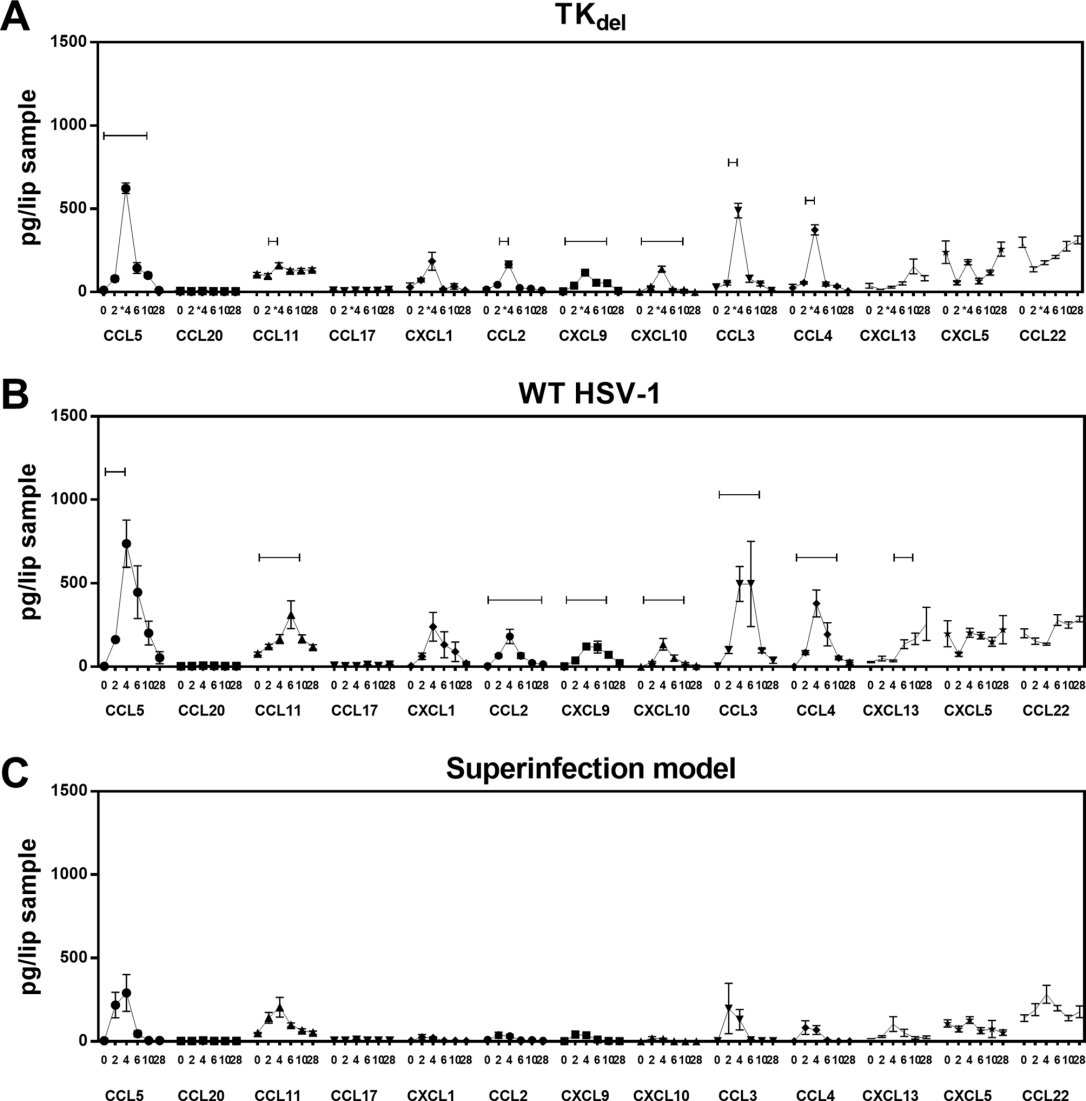
Inflammatory chemokines at the lip site of inoculation. Naïve mice were infected in the lip with TK_del_ (**A**), or WT HSV-1 (**B**) on day 0. Mice of the superinfection model were initially inoculated with TK_del_ in the right lip on day -4 and then superinfected with WT HSV-1 on day 0 in the left lip (**C**). Mice were randomly culled at indicated time points and inflammatory chemokines in non-denatured lip tissue lysates were quantified using multi-analyte flow cytometry assay. Bars indicate significant difference from uninfected day 0 (*p* < *0.03; T-test*). Each point represents *n* = *4* independent biological replicates. *Figures were formulated using LEGENDplex software, Excel and Prism.*

The dynamics of inflammatory chemokines at the site of challenge with either TK_del_ or WT HSV-1 were quite similar, with CCL3, 4 and 5 increasing the most, followed by CCL11 and 2, and CXCL9 and 10 (Fig. 6A,B). While there were trends of increased CCL5, 11, 3 and 4, at the site of virulent WT-challenge in TK_del_-primed mice, there was no significance compared to day 0 levels (Fig. 6C).

### Contralateral lip immune conditioning after lytic TK_del_ lip prime

**Figure 7:**
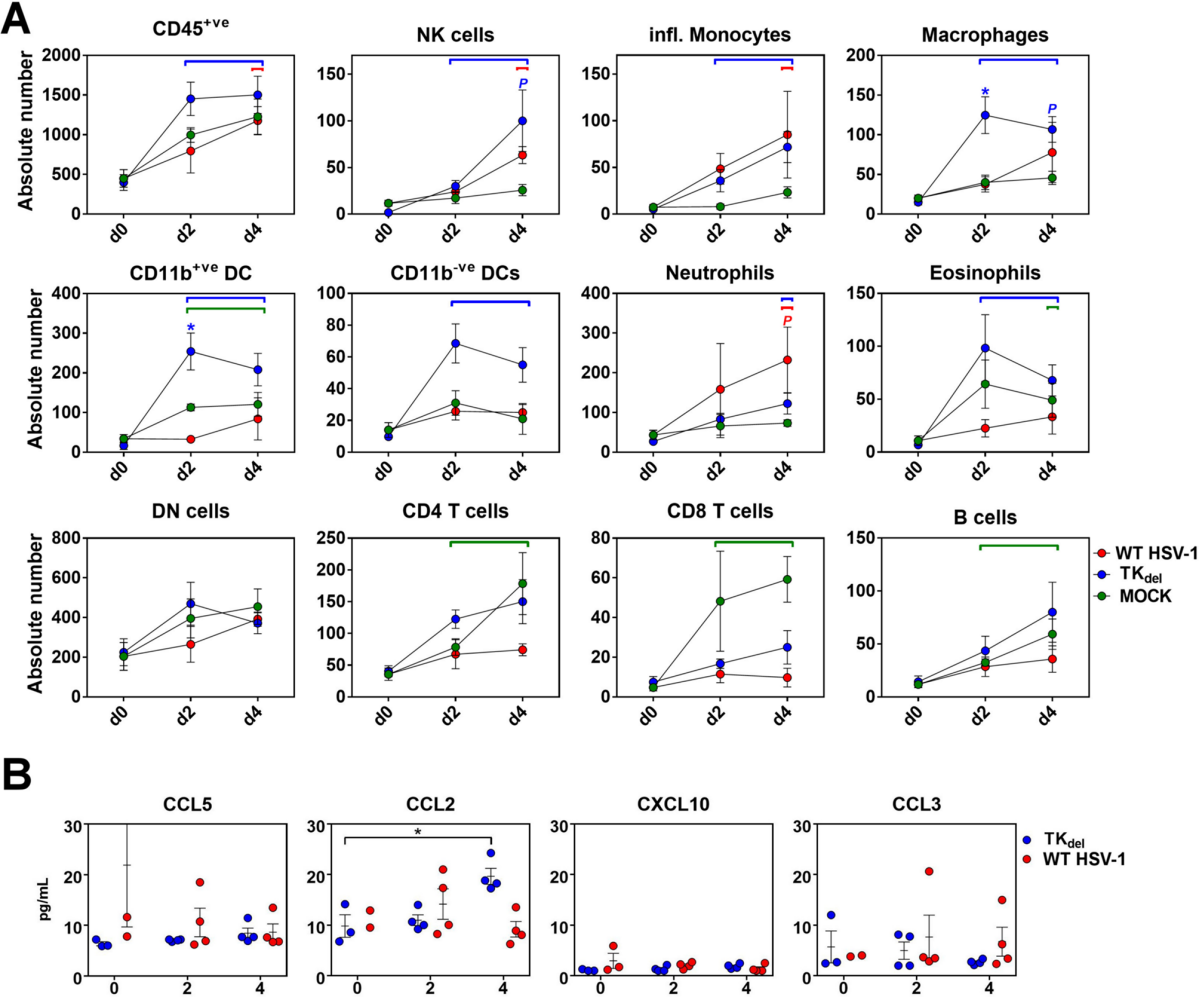
Immune subsets and inflammatory chemokines in the lip contralateral to prime. Naïve mice were infected with WT HSV-1, PBS (Mock), or TK_del_. On different days for each group, immune subsets were defined and absolute numbers enumerated from single cell suspensions by flow cytometry from lip tissue contralateral to the inoculation site (**A**), or inflammatory chemokines were quantified in non-denatured lip tissue lysates (**B**). *Colored bars* indicating significant difference from uninfected tissue on day 0. *Blue asterisk* indicates a significant difference for TK_del_ compared to PBS mock infection (green) and WT HSV-1 (*p* < *0.03; permutation test*). *Colored P* indicates a significant difference between the color-corresponding group and the Mock group. *Black asterisks* indicate a significant difference (*p* < *0.03; T-test*). Each point represents *n* = *4* individual biological replicates. *Figures were formulated using Flowjo, Excel and Prism.*

During the window of time from prime with TK_del_ in the right lip, to before challenge with virulent WT in the left lip on day 4, CD45^+ve^ cells had increased in the left lip that is contralateral to the TK_del_ prime (Fig. 7A). NK cells, inflammatory monocytes, macrophages, CD11b^−ve^ and CD11b^+ve^ DC, and neutrophils had all increased in lip contralateral to either TK_del_ or WT HSV-1 inoculation by day 2 and 4, and in contrast to mock infection (Fig. 7A). From the panel of inflammatory chemokines quantified (Figs. 3 and 6), CCL2 was the only chemokine elevated, by day 4 post prime with TK_del_ (Fig. 7B; *all other chemokines were tested, but did not demonstrate increases, as exemplified by CCL5, CXCL10 and CCL3*). Therefore, primary HSV-1 lip infection induces a heightened state of immunosurveillance in the contralateral lip, with some subtle differences occurring by day 4.

## Discussion

Priming mice with non-neurovirulent TK_del_ elicits protection against contralateral challenge 4 days later, to eliminate acute ocular disease, reporter gene expression, and reactivation from latent state when triggered by explant culture^30^. Priming in the lip with TK_del_ ‘conditioned’ the contralateral lip (the prospective site of superinfection challenge), before the superinfection occurs. Elevated CCL2 and numbers of NK and DC cells infiltrated in the days subsequent to prime on the same day that the protective state is established. This signature demonstrates the potential for enhanced immune responsiveness, facilitating recruitment of CCR2^+ve^ cells to the challenge site earlier, as described for many disease states^39^. Within hours of sensing HSV-1, NK cells can respond to elicit cytolytic control of infection^17,20,40^. Thus, these augmented local immune mechanisms prior to virulent WT challenge likely attenuate viral gene expression, to play an essential role in the rapid viral control after priming. The most commonly used strain of mouse used for HSV-1 immunology studies, C57BL/6, bears a natural resistance to neurological spread that is mediated by NK1.1^+ve^ NK cells^41^. BALB/c mice lack this NK subset, which and are thus burdened with delayed responsiveness and weakened control of acute HSV-1 infection^42^. Therefore, heightened immunosurveillance in TK_del_-primed mice may overcome this delayed immune responsiveness upon challenge, and should be considered as potential critical aspect in establishing the acute disease-free and/or non-reactivating latent HSV-1 reservoirs in our model^30^.

CXCL11, CXCL10, and CXCL9 all signal through the CXCR3 receptor^43^, to attract T cells and NK cells to sites of HSV-1 infection^44,45^. CXCL9 and CXCL10 are produced as a TLR9/interferon alpha-mediated response to inoculation of the cornea with HSV-1 via corneal scarification^46^. We detected CXCL10 and CXCL9 surges during primary infection to mirror NK cell, neutrophil and monocyte influxes. In the presence of interferon gamma (IFNγ) and HSV-1, neutrophils are a potent producer of CXCL9 and CXCL10^47^, and their presence in combination with NK cells that produce IFNγ in response to HSV-1^48^ could explain the massive increases that were seen in challenged naïve animals, but were absent in primed animals that lacked major neutrophil influxes when challenged. Even so, CXCL9 and CXCL10 presence did not mirror T cell infiltration, which occurred by day 6; the naïve status of unprimed mice and the lack of primed T cells helps to explain their early absence. However, it is also possible that CXCL11 (not included in our study) was involved in T cell recruitment at these later time points^49^.

While 60–75% of the peripheral T cells that recognize HSV-1 in C57BL/6 mice are raised to a single epitope of the true late gB protein^50^ with some diversity in subdominant responses^51^, the repertoire diversity of BALB/c mice recognising HSV-1 remains undefined. In C57BL/6 mice, inoculation of HSV-1 into skin elicits a circulating lytic CD8 T cell response to HSV-1 gB within 4 days^52^, and if present within the first 24 h of infection it can terminate an establishing acute infection^22^. However, infection of iTG neurons occurs within hours after primary infection in the lip in our model and in others^25,53–55^. Priming with TK_del_ led to a more rapid influx of CD4 and CD8 T cells into the WT-challenged lip, being detected by the first time point (day 2) while T cell influx in naïve mice occurred by days 4–6. A 4-day delay is also observed for the establishment of protection after TK_del_ prime^30^. The already defined role of T cells in extinguishing HSV-1 infection, and their enhanced kinetics in our model to coincide with protection, suggests they are undoubtedly involved in establishing the acute disease-free, non-reactivating latent state.

B cells also play a major role, elicited by live viral vaccine to recognise numerous capsid and envelope proteins in reducing or eliminate viral replication, and corneal defects and scarring after corneal challenge^56^. This protection was independent of complement and FcγRIII. B cells have numerous roles during acute infection, from performing effector functions, secretion of antibodies, to antigen presentation^57^. We detected subtle increasing CXCL13 (recruitment factor for B cells^58^) and B cell trends at sites of HSV-1 prime or challenge, to suggest B cell responses may also be involved in local protection, and a combined enhanced T cell and B cell response are elicited by live-attenuated vaccine strategies^59^. However, it remains to be shown if B cell responses are also involved in limiting reactivation events. In our previous study, footpad prime with TK_del_ instead of in the lip elicited protection against acute OHD development upon virulent WT challenge in the lip, but reactivation events still occurred from the iTG^30^. In contrast, the lip prime with TK_del_ elicited protection to both.

The innate inflammatory response is a consequence of lytic HSV-1 infection, driven by the sensing of virus and tissue damage^60,61^. Inflammatory chemokines are also responsible for recruitment of specific immune subsets that can exacerbate HSV-1 disease in the cornea during lytic infection^62–64^. The iTG of naïve unprimed mice developed a bold inflammatory response starting 2 days after WT challenge in the lip, consisting of inflammatory chemokines, inflammatory monocytes, neutrophils, macrophages, DC and NK cells. While these cell populations have demonstrated anti-HSV-1 properties^13,15–21,65,66^, most can also be responsible for exacerbated inflammation and cause neurological damage during their responses^39,62,67–71^. It remains unclear if this increased inflammatory response influences latent reservoir establishment in regards to reactivation. Mice primed with TK_del_ lacked an exacerbated inflammatory during acute phase WT-challenge in the iTG, with a clear lack of innate inflammatory subsets (macrophages, neutrophils, eosinophils and CD11b^+ve^ DC) and attenuated inflammatory chemokines. The concordant absence of detectable WT HSV-1 gene expression during acute phase of WT-challenge suggests that lytic replication within the iTG is tempered by priming with TK_del_, and limits viral spread to additional neurons, such as those that innervate the cornea^28,29,35,72^.

HSV-1 latent state is defined solely by the absence of infectious virus particle production^27^, which occurs by day 28 in our model^29,35,72^. Many different mechanisms are implicated in the interdiction of HSV-1 replication to maintain latency, a state not considered as inert (‘attempted’ replication may occur continually or sporadically)^60,73–76^. Classically, clonal T cells persist in latent TG reservoirs, a phenomenon involving local T cell epitope presentation^77–79^, eliciting responses that in turn are supposed to inhibit reactivation^80–83^. However, the role of T cells still remains unclear as a recent study of their depletion during induced reactivation did not influence reactivation events^84^.

CD8 T cell-derived CCL5 is important for the retention of CD4 T cells, at least within the skin after HSV infection^43,85,86^. In our model, CCL5 production was high during infection of naïve mice, remaining elevated during latent infection, but was drastically reduced when TK_del_-primed mice were challenged with WT. In these TK_del_-primed mice, T cell accumulation is lost from latent TG reservoirs, signalling a major loss of viral epitope presentation and cessation of immune stimulation^63^. The further inability of HSV-1 to reactivate from WT-challenged TK_del_-primed mouse TG, and the attenuated promoter activity of latent reservoirs^30^, supports the notion that the proposed ‘dead-end’ latent state is unlike the typical latent state that establishes in naïve mice as it appears more inert. Local TK_del_ primary infection is essential for this enhanced protection, which may be facilitated by enhanced kinetics of the immune response, but also by skewing the T cells repertoire or functionality (due to inherent properties of TK_del_). This notion may be important in HSV-1 immunity and is supported by the presence of pathogenic vs protective T cell responses^87–89^, which are elicited during primary antigen exposure^90^.

To summarize, orofacial prime with non-neurovirulent TK_del_ elicited enhanced contralateral immunosurveillance before virulent WT HSV-1 challenge in the contralateral lip. Attenuated inflammatory responses and lymphocyte-enriched infiltrate in the WT challenge inoculation site and iTG during acute infection phase correlated with subdued or undetectable viral gene expression. Even though the skin is a protective layer designed to take the brunt of a viral infection and initiate adaptive immune responses, HSV-1 spreads rapidly to iTG neurons. Thus, protective immune mechanisms may need to be established locally, in orofacial skin, for ‘dead-end’ latent state to be achieved in the iTG. Investigations are continuing to identify how the enhanced immunosurveillance, adaptive immune responses, and TK_del_ properties are responsible for establishing a ‘dead-end’ latent state. Defining the immune phenomena that occur during the establishment of reactivating versus non-reactivating HSV-1 latent reservoirs is essential for vaccine development, to be exploited for the prevention of disease caused by HSV-1 reactivation.

## Supplementary Information

Supplementary Figures.

## Data Availability

The datasets generated and/or analysed during the current study are available in FlowRepository, http://flowrepository.org/id/FR-FCM-Z4VN.
