# Supplementary material for: Initial TK-deficient HSV-1 infection in the lip alters contralateral lip challenge immune dynamics
Source: Sci Rep. 2022 May 19;12:8489. doi: 10.1038/s41598-022-12597-4 (PMC9119387; doi:10.1038/s41598-022-12597-4)
Supplement: Supplementary file 1 — Supplementary Figures. [file 41598_2022_12597_MOESM1_ESM.pdf]

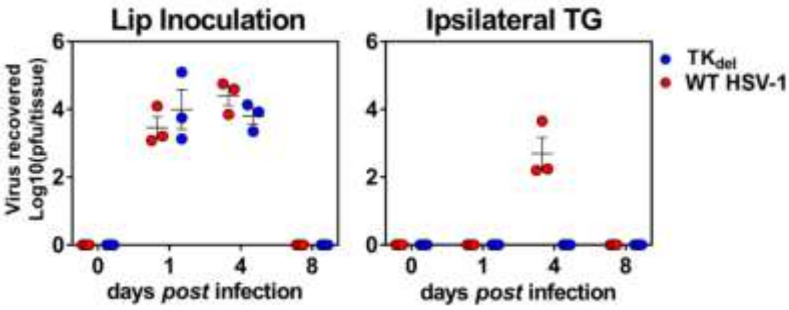

Supplementary Figure 1. Recovery of infectious virus from sites of HSV-1 acute infection. Groups of naïve mice (*n* = *12*) were infected in the lip with 1 x 10^6 PFU (in 1 µL) of either SC16 or TK_{del}. On different days during acute phase, the site of inoculated lip or whole iTG (*n* = *3/group*) were harvested and infectious particles liberated by non-denaturing mortar and pestle grinding and PBS dilution. Lysates were used to infect VERO monolayers and plaques enumerated.

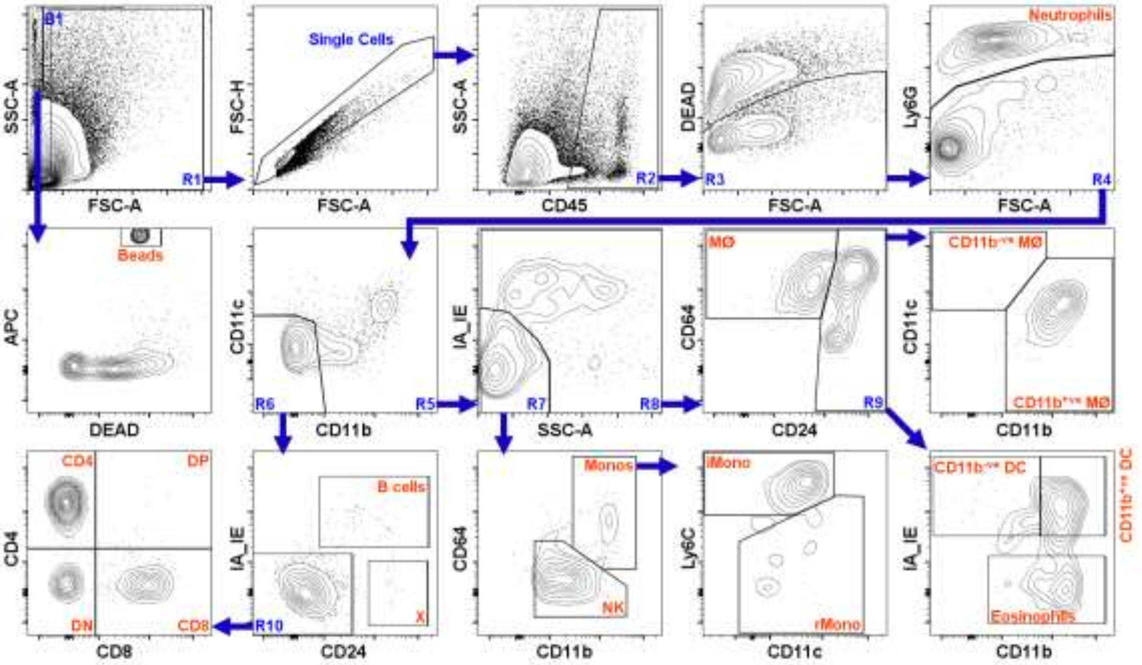

Supplementary Figure 2. Characterization of immune infiltrate into HSV-1-infected tissue using a modified gating strategy previously defined by Yu et al. 2016. Single cell suspension of stained SC16-infected mouse whole iTG was gated to identify: Trucount beads (Beads), or Single cells, $CD45^{+ve}$ cells (R2), live cells (R3), $Ly6G^{+ve}$ Neutrophils, or $Ly6G^{-ve}$ R4, $CD11b^{-ve}$ $CD11c^{-ve}$ cells (B cells and CD4+ve or CD8+ve T cells), or $CD11b^{+ve}$ and $CD11c^{+ve}$ cells (R5). MHC class II^{-ve} and SSC^{lo} cells (R7) were separated by CD64 and CD11b to define NK and monocytes. Remaining MHC class II^{+ve} and/or $SSC^{mid/hi}$ cells were separated into macrophages and DC subsets based on a combination of CD64 or $CD24^{+/-}$ CD64, respectively. Eosinophils were separated from $CD11b^{+ve}$ or $CD11b^{-ve}$ DC by the lack of MHC class II expression from gate R9, and were $CD11b^{+ve}$.
